# The human type 2 diabetes-specific visceral adipose tissue proteome and transcriptome in obesity

**DOI:** 10.1038/s41598-021-96995-0

**Published:** 2021-08-30

**Authors:** Nicholas J. Carruthers, Clarissa Strieder-Barboza, Joseph A. Caruso, Carmen G. Flesher, Nicki A. Baker, Samuel A. Kerk, Alexander Ky, Anne P. Ehlers, Oliver A. Varban, Costas A. Lyssiotis, Carey N. Lumeng, Paul M. Stemmer, Robert W. O’Rourke

**Affiliations:** 1 Proteomics Core Facility, Wayne State University, 42 W. Warren Ave, Detroit, MI 48202 USA; 2 Department of Surgery, University of Michigan Medical School, 1301 Catherine St, Ann Arbor, MI 48109 USA; 3 Department of Chemistry, University of Michigan Medical School, 1301 Catherine St, Ann Arbor, MI 48109 USA; 4 Department of Molecular and Integrative Physiology, University of Michigan Medical School, 1301 Catherine St, Ann Arbor, MI 48109 USA; 5 Division of Gastroenterology, Department of Internal Medicine, University of Michigan Medical School, 1301 Catherine St, Ann Arbor, MI 48109 USA; 6 Rogel Cancer Center, University of Michigan Medical School, 1301 Catherine St, Ann Arbor, MI 48109 USA; 7 Department of Pediatrics and Communicable Diseases, University of Michigan Medical School, 1301 Catherine St, Ann Arbor, MI 48109 USA; 8 Graduate Program in Immunology, University of Michigan Medical School, 1301 Catherine St, Ann Arbor, MI 48109 USA; 9 Graduate Program in Cellular and Molecular Biology, University of Michigan Medical School, 1301 Catherine St, Ann Arbor, MI 48109 USA; 10 Department of Surgery, Veterans Affairs Ann Arbor Healthcare System, 2215 Fuller Rd, Ann Arbor, MI 48105 USA; 11 Section of General Surgery, Department of Surgery, University of Michigan, 2210 Taubman Center-5343, 1500 E. Medical Center Drive, Ann Arbor, MI 48109-5343 USA

**Keywords:** Diseases, Diabetes, Obesity

## Abstract

Dysfunctional visceral adipose tissue (VAT) in obesity is associated with type 2 diabetes (DM) but underlying mechanisms remain unclear. Our objective in this discovery analysis was to identify genes and proteins regulated by DM to elucidate aberrant cellular metabolic and signaling mediators. We performed label-free proteomics and RNA-sequencing analysis of VAT from female bariatric surgery subjects with DM and without DM (NDM). We quantified 1965 protein groups, 23 proteins, and 372 genes that were differently abundant in DM vs. NDM VAT. Proteins downregulated in DM were related to fatty acid synthesis and mitochondrial function (fatty acid synthase, FASN; dihydrolipoyl dehydrogenase, mitochondrial, E3 component, DLD; succinate dehydrogenase-α, SDHA) while proteins upregulated in DM were associated with innate immunity and transcriptional regulation (vitronectin, VTN; endothelial protein C receptor, EPCR; signal transducer and activator of transcription 5B, STAT5B). Transcriptome indicated defects in innate inflammation, lipid metabolism, and extracellular matrix (ECM) function, and components of complement classical and alternative cascades. The VAT proteome and transcriptome shared 13 biological processes impacted by DM, related to complement activation, cell proliferation and migration, ECM organization, lipid metabolism, and gluconeogenesis. Our data revealed a marked effect of DM in downregulating FASN. We also demonstrate enrichment of complement factor B (CFB), coagulation factor XIII A chain (F13A1), thrombospondin 1 (THBS1), and integrins at mRNA and protein levels, albeit with lower q-values and lack of Western blot or PCR confirmation. Our findings suggest putative mechanisms of VAT dysfunction in DM.

## Introduction

Obesity and associated metabolic diseases, including type 2 diabetes mellitus (DM), is a major public health challenge. By 2030, the global incidence of DM is estimated to reach 350 million cases, accounting for > 70% of early deaths worldwide. An understanding of mechanisms linking obesity to metabolic disease is of critical importance.

Adipose tissue plays a key role in the pathogenesis of DM, evidenced by data demonstrating that selective enhancement of adipose tissue insulin sensitivity ameliorates systemic insulin resistance in murine obesity^1^. Visceral adipose tissue (VAT) plays a particularly important role in DM, as increased visceral adiposity is highly correlated with insulin resistance in patients with DM^2^. Dysfunctional VAT in DM is characterized by adipocyte hypertrophy and cellular insulin resistance, changes in extracellular matrix (ECM) composition and function, and increased infiltration of inflammatory immune cells^3–5^. VAT is therefore a key target organ in which novel molecules and pathways linked to DM pathogenesis can be identified.

Proteomic studies of human adipose tissues provide an understanding of aberrant signaling pathways associated with DM^6–9^. Kim et al. performed nano-liquid chromatography-mass spectrometry (LC–MS)/mass spectrometry (MS) analysis of human VAT and generated data suggesting cross-talk among VAT cells associated with early DM pathogenesis^7^. Fang et al. used LC–MS to survey the proteome of matched subcutaneous and visceral adipose tissues from patients with and without DM and identified protein targets associated with DM, including but not limited to moesin, GRP78, and cytochrome c oxidase subunit 6B1^9^. These studies and others demonstrate that proteomic analysis of adipose tissue can identify signaling proteins involved in DM pathogenesis. Comprehensive studies integrating adipose tissue proteome and RNA sequencing data simultaneously, however, could provide a better understanding of biological processes and signaling mediators regulating pathogenesis of metabolic disease. Here, we used label-free proteomics and RNA sequencing analyses of VAT from female patients with obesity with and without DM, with the goal of identifying genes and proteins regulated by DM at both transcriptome and proteome levels as high yield targets for elucidating aberrant cellular metabolic and signaling mediators associated with DM. Our findings elucidate potential mechanisms of adipose tissue dysfunction associated with DM pathogenesis.

## Results

### VAT proteome: depth of analysis, differentially expressed proteins

**Figure 1:**
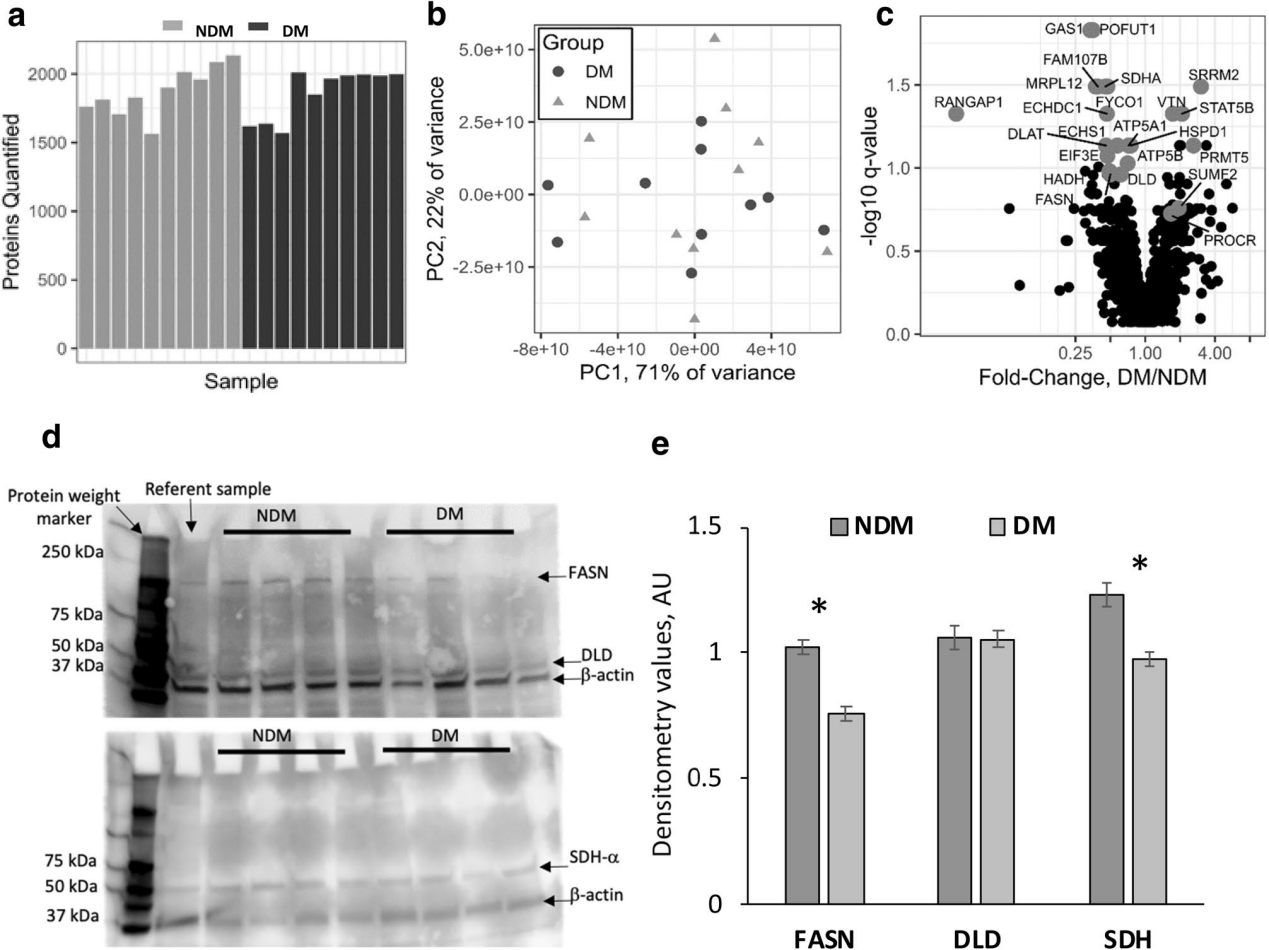
Sample preparation and differentially abundant proteins in DM and NDM tissues. Sample preparation was reproducible and similar between groups, as determined by the number of proteins quantified (**a**) and principal component analysis (**b**); no significant difference between groups was observed in number of proteins quantified; principal component projection does not separate DM from NDM samples indicating that overall protein composition of samples is similar between groups. (**c**) Volcano plots of protein abundance in DM compared with NDM samples. Light grey dots indicate differentially abundant proteins between groups; black dots represent proteins that were not significantly regulated. Labels indicate gene symbols. (**d**) Western blots for select targets differentially regulated in proteomic analysis. (**e**) Western blotting densitometry quantification. *p-value < 0.001, independent t-test comparing NDM (n = 8) to DM (n = 8) densitometry values, normalized to actin as described in methods.

To identify metabolic and signaling disruptions in adipose tissue associated with DM, we conducted proteomic analysis of human VAT from 10 NDM and 10 DM age- and BMI-matched female patients with obesity. 2640 proteins were identified in total, of which 1965 had sufficient data for quantitative analysis, defined as being quantified in at least 4 samples in each group (Supplementary Tables S1, S2). The number of quantified proteins was similar between DM and NDM samples, confirming effective sample preparation for both groups (Fig. 1a). Principal component analysis confirmed overall similarity between groups, which could not be separated by the first two principal components (92% of variance total, Fig. 1b). Twenty-three proteins were differentially abundant between groups, among which 6 were upregulated and 17 were downregulated in DM compared to NDM VAT (Fig. 1c, Table 1). Proteins upregulated in DM were primarily associated with innate immunity and transcriptional regulation; downregulated proteins were related to fatty acid synthesis and detoxification, and mitochondrial function.

**Table 1:** Differentially abundant proteins in DM compared with NDM patients.

Protein accession number	Protein name, corresponding gene	Fold change DM/NDM	q-value^a^	Pass rate^b^	Protein function
Q9UQ35	Serine/arginine repetitive matrix protein 2 (*SRRM2*)	3.03	0.032	0.62	Pre-mRNA Splicing
O14744	Protein arginine *N*-methyltransferase 5 (*PRMT5*)	2.61	0.073	0.66	Protein methylation
P51692	Signal transducer and activator of transcription 5B (*STAT5B*)	2.08	0.047	0.66	Transcriptional activating factor
Q8NBJ7	Sulfatase-modifying factor 2 (*SUMF2*)	1.95	0.174	0.84	Implicated in metabolism
P04004	Vitronectin (*VTN*)	1.73	0.047	1	Innate immunity
Q9UNN8	Endothelial protein C receptor (*EPCR*)	1.67	0.188	0.88	Innate immunity, coagulation
P10809	60 kDa heat shock protein, mitochondrial (*HSPD1*)	0.75	0.073	1	Protein folding, mitochondrial protein import
P25705	ATP synthase subunit alpha, mitochondrial (*ATP5F1A*)	0.72	0.073	1	Oxidative phosphorylation
P06576	ATP synthase subunit beta, mitochondrial (*ATP5F1B*)	0.71	0.094	0.96	Oxidative phosphorylation
P09622	Dihydrolipoyl dehydrogenase, mitochondrial, E3 component (*DLD*)	0.62	0.109	0.62	Component of pyruvate dehydrogenase complex
P30084	Enoyl-CoA hydratase, mitochondrial (ECHS1)	0.58	0.073	1	Fatty acid synthesis
P49327	Fatty acid synthase (*FASN*)	0.5	0.109	0.68	Fatty acid synthesis
Q16836	Hydroxyacyl-coenzyme A dehydrogenase, mitochondrial (*HADH*)	0.49	0.105	0.76	3-Hydroxyacyl-CoA oxidation
Q9BQS8	FYVE and coiled-coil domain-containing protein 1 (*FYCO1*)	0.47	0.032	0.8	Microtubule plus end-directed vesicle transport
P60228	Eukaryotic translation initiation factor 3 subunit E (*EIF3E*)	0.47	0.085	0.96	Protein synthesis
Q9NTX5	Ethylmalonyl-CoA decarboxylase (*ECHDC1*)	0.47	0.047	1	Fatty acid metabolite detoxification
P10515	Dihydrolipoyllysine-residue acetyltransferase component, pyruvate dehydrogenase complex, mitochondrial, E2 component (*DLAT*)	0.46	0.073	1	Component of pyruvate dehydrogenase complex
P31040	Succinate dehydrogenase [ubiquinone] flavoprotein subunit, mitochondrial (*SDHA*)	0.45	0.032	1	SDH complex activity, TCA cycle, Oxidative phosphorylation
Q9H098	Protein FAM107B (*FAM107B*)	0.39	0.032	0.74	Homologous to cytoskeletal regulators in Rho family
P52815	39S ribosomal protein L12, mitochondrial (*MRPL1*)	0.38	0.032	1	Mitochondrial translation
Q9H488	GDP-fucose protein *O*-fucosyltransferase 1 (*POFUT1*)	0.35	0.015	0.96	Glycosyltransferase, NOTCH signaling
P54826	Growth arrest-specific protein 1 (*GAS1*)	0.34	0.015	0.68	Cell cycle growth suppression
P46060	Ran GTPase-activating protein 1 (*RANBP1*)	0.02	0.047	0.94	Regulates nuclear-transport

^a^FDR corrected p-value^50^ for differential abundance between DM and NDM tissues (moderated t-test, n = 10).

^b^Fraction of imputations where this protein was significant (q < 0.1).

We validated protein expression of three proteins identified as differentially regulated based on MS analysis in VAT samples from 4 NDM and 4 DM age- and BMI-matched female patients using Western blot analysis. We confirmed decreased protein abundance of fatty acid synthase (FASN) and succinate-ubiquinone oxidoreductase subunit α (SDH-α), but not dihydrolipoamide dehydrogenase (DLD), in VAT from DM relative to NDM subjects (Fig. 1d,e). Using heuristic testing, proteomics data demonstrate that the complement component C1q receptor was uniquely expressed in DM samples, while other 10 differentially regulated proteins associated with mitochondrial function and TCA cycle were uniquely expressed in NDM samples (Supplementary Table S3).

### Differentially abundant protein categories: protein set enrichment analysis

**Figure 2:**
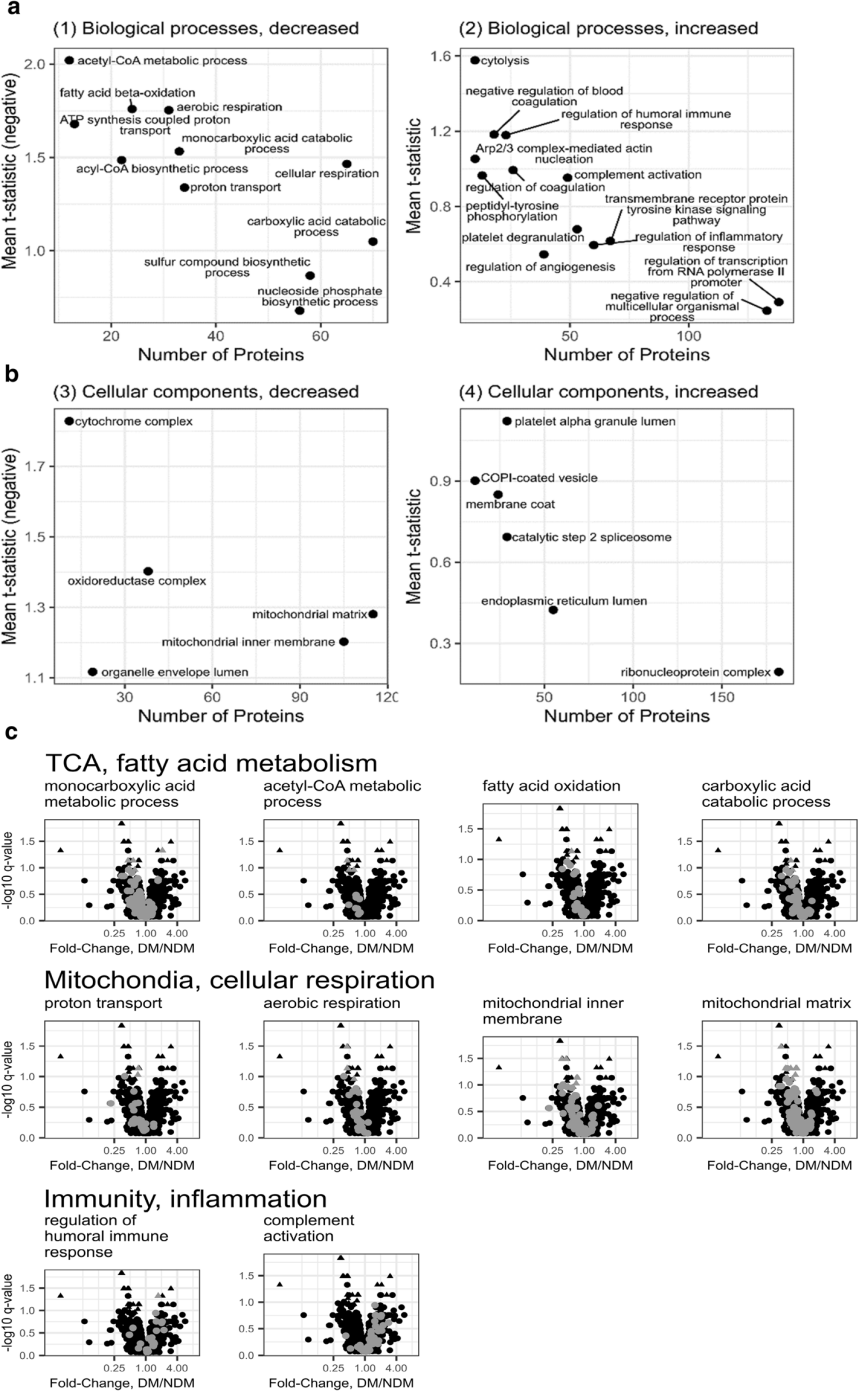
Protein categories of biological processes and cellular components affected by DM. Biological processes (**a**) and cellular components (**b**) decreased (**a1**,**b3**) or increased (**a2**,**b4**) in abundance in DM samples relative to NDM analyzed by PIANO (10% FDR). X-axis represents the number of proteins in each category; y-axis represents the value for a given category analyzed by enrichment test-statistic and related to mean of t-statistics for individual in-category proteins. Categories in lower right quadrants have multiple proteins with small abundance differences between DM and NDM groups, while categories in upper left quadrants have fewer proteins with larger abundance differences between DM and NDM groups. Categories displayed are a subset of all affected categories selected to minimize redundancy. (**c**) Differentially regulated pathways: Volcano plots of protein abundance for categories affected by DM. Protein abundance in categories related to TCA cycle, fatty acid metabolism, mitochondria, and cellular respiration were decreased, and immunity and inflammation proteins were increased in DM (PIANO, 10% FDR). Light grey points indicate proteins that are category members, triangles indicate proteins significantly regulated between DM and NDM (limma, q < 0.1).

T-statistics and identifiers for all quantified proteins were submitted to PIANO software for protein set enrichment analysis to identify protein categories with differential expression between groups. 120 biological processes and 28 cellular components were increased, and pathways for 121 biological processes and 20 cellular components, were decreased in DM relative to NDM tissue (Supplementary Table S4). Identified categories that were affected by DM included a range of values for the number of proteins identified and response magnitude as measured by the mean t-statistic (Fig. 2a,b). For example, acetyl-CoA metabolic process (GO:0006084) contained 12 proteins with a mean t-statistic of -2.0 while mitochondrial matrix (GO:0005759) contained 115 proteins with a mean t-statistic of − 1.3 (Fig. 2a). Categories with decreased abundance in DM samples relative to NDM are primarily related to mitochondria, including biological process categories related to ATP synthesis, cellular respiration, and acyl-CoA and fatty acid metabolism and subcellular location categories including both membrane-bound and luminal components (Fig. 2a,b). The decreased abundance of multiple distinct mitochondrial categories in DM tissues relative to NDM indicates that decreased abundance of mitochondrial proteins is a robust finding. Notable among categories with increased abundance in DM are proteins related to humoral immune response and platelet degranulation (Fig. 2a,b, Supplementary Table S4). Volcano plots highlighting protein categories related to the Tricarboxylic Acid Cycle (TCA) and fatty acid metabolism, mitochondria and cellular respiration, and immunity and inflammation show coordinated shifts of large fractions of proteins in each category (Fig. 2c). For example, nearly all proteins in the carboxylic acid catabolic process category are shifted to the left, reflecting decreased abundance in DM tissues relative to NDM. These plots illustrate a clear impact of DM on identified categories even while the number of individual proteins in any category that were significantly affected may be small.

### Differentially expressed genes identified by RNA sequencing of VAT

**Figure 3:**
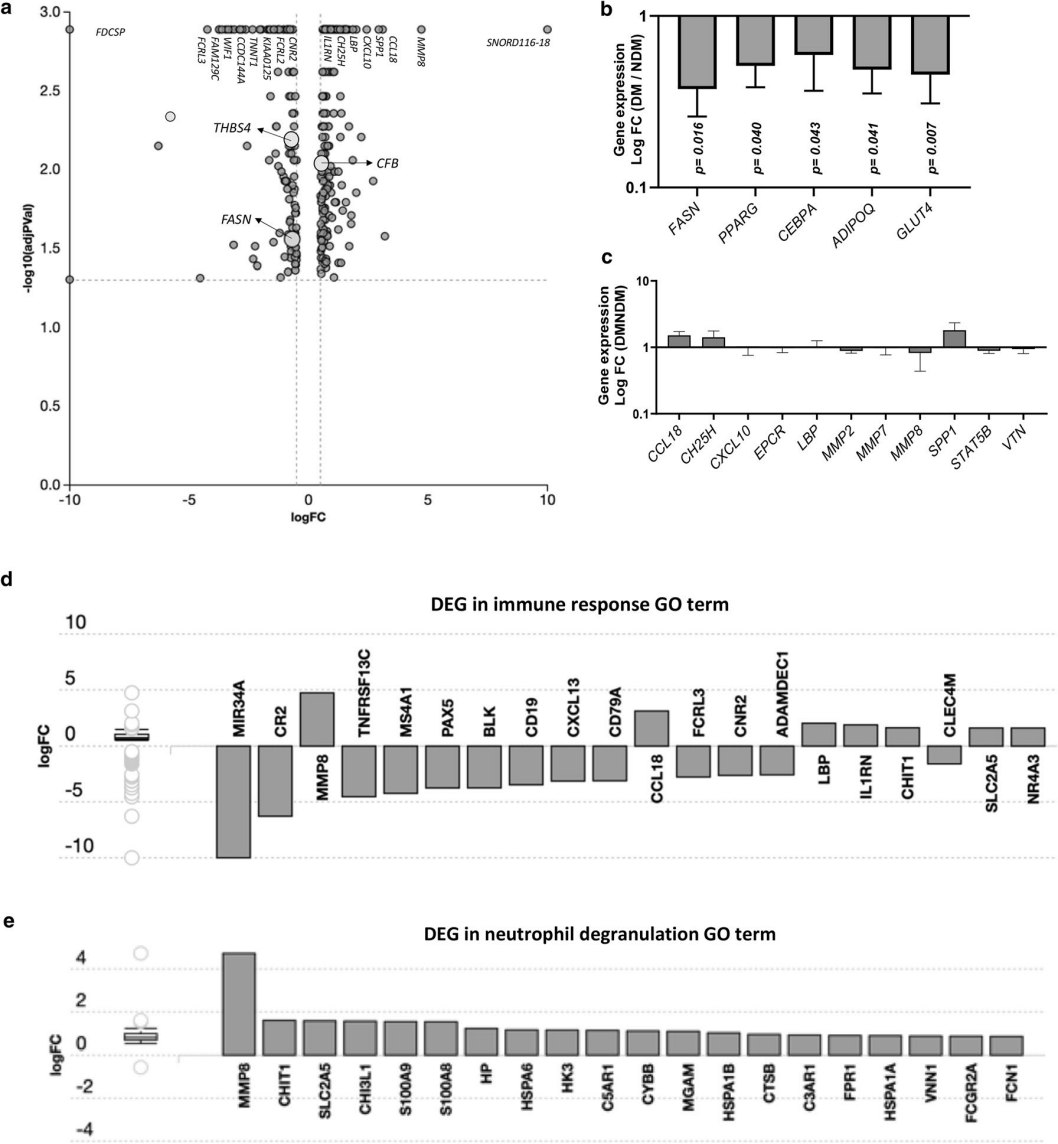
Differentially expressed genes and biological processes in DM and NDM VAT. (**a**) Volcano plot of gene expression in DM compared with NDM samples. All 372 significantly expressed genes (DEG) are represented in terms of their measured expression change (x-axis; log fold change) and the significance of the change (y-axis). The significance is represented as negative log (base 10) of the p-value, so that more significant genes are plotted higher on the y-axis. Genes represented on the left of logFC = 0 in the x-axis were downregulated in DM, and genes represented on the right were upregulated in DM. Dotted lines represent the thresholds used to select the DEG: 0.5 for expression change and 0.05 for significance. FASN, CFB and THBS-4 are highlighted in graph as molecules significantly regulated by DM at mRNA and protein levels. (**b**) qRT-PCR analysis of VAT for *FASN* gene expression and other markers of adipogenesis, lipogenesis. Results shown as log fold change in DM compared to NDM samples. (**c**) qRT-PCR analysis of VAT for inflammatory gene expression. Results shown as log fold change in DM compared to NDM samples, all p > 0.050. (**d**,**e**) Top 20 DEG genes in top scoring biological processes in obese human VAT. DEG are ranked based on absolute value of log fold change. The box and whisker plot on left summarizes the distribution of all DEG in this GO term. The box represents 1st quartile, median and 3rd quartile, while outliers are represented by circles. Bars bellow logFC = 0 represent genes downregulated in DM compared to NDM samples, while bars above logFC = 0 represent genes upregulated in DM compared to NDM patients.

We performed RNA sequencing analysis of VAT samples from 5 NDM and 5 DM age- and BMI-matched female patients with obesity. RNA sequencing of VAT identified 372 differentially expressed genes (DEG) between DM and NDM samples out of a total of 17,753 genes with measured expression (Fig. 3a). The top 20 differentially upregulated or downregulated genes in DM compared with NDM patients are shown in Table 2. Among these DEG, upregulation of C–C motif chemokine ligand 18 (*CCL18),* osteopontin *(SPP1),* C-X-C motif chemokine ligand 10 *(CXCL10),* lipopolysaccharide binding protein *(LBP),* cholesterol 25-hydroxylase *(CH25H),* interleukin 1 receptor antagonist *(IL1RN),* matrix metalloprotease 8 (*MMP7),* and matrix metalloprotease 8 (*MMP8)* in DM VAT highlights an increased inflammatory response linked to defective lipid metabolism and ECM function in obese DM VAT (fold changes for these DEG shown in Table 2). These DEG form part of chemokine signaling, cytokine–cytokine receptor interaction, IL17 signaling, and ECM-receptor interaction pathways, which were among the 25 most significantly impacted pathways by DM, considering FDR correction (Supplementary Table S5). Additionally, upregulation of *SPP1, CD19, CH25H*, and *IL1RN* are associated with alterations in regulation of lipid metabolic process, a GO term significantly affected by DM as revealed by both proteomic and transcriptomic analyses (Supplementary Table S6). Notably, among 23 proteins identified as differentially abundant between DM and NDM VAT (Fig. 1c, Table 1), only *FASN* was also identified as a DEG by RNA sequencing (Fig. 3a). Both proteomic and transcriptomic analyses identified FASN as being downregulated in DM vs. NDM VAT (RNAseq: LogFC = − 0.684; p-value = 0.028).

**Table 2:** Top 20 differentially regulated genes identified by RNA sequencing analysis of human obese VAT comparing DM with NDM patients. Significance represented as negative log (base 10) of p-value.

Upregulated in DM				Downregulated in DM			
Gene name	Gene symbol	LogFC	p-value	Gene name	Gene symbol	LogFC	p-value
Small nucleolar RNA, C/D box 116-18	*SNORD116-18*	10.00	0.001	Follicular dendritic cell secreted protein	*FDCSP*	− 10.00	0.001
Matrix metallopeptidase 8	*MMP8*	4.723	0.001	Microrna 34a	*MIR34A*	− 10.00	0.050
Matrix metallopeptidase 7	*MMP7*	3.194	0.027	Complement C3d receptor 2	*CR2*	− 6.286	0.007
C–C motif chemokine ligand 18	*CCL18*	3.102	0.001	TNF receptor superfamily member 13C	*TNFRSF13C*	− 4.536	0.049
Secreted phosphoprotein 1	*SPP1*	2.939	0.001	Membrane spanning 4-domains A1	*MS4A1*	− 4.235	0.001
Ring finger protein 128	*RNF128*	2.706	0.012	Paired box 5	*PAX5*	− 3.744	0.001
C-X-C motif chemokine ligand 10	*CXCL10*	2.436	0.001	BLK proto-oncogene, Src fam tyrosine kinase	*BLK*	− 3.737	0.001
Ribosomal protein S4 Y-linked 1	*RPS4Y1*	2.208	0.006	Fc receptor like A	*FCRLA*	− 3.626	0.001
Lipopolysaccharide binding protein	*LBP*	2.000	0.001	CD19 molecule	*CD19*	− 3.466	0.001
Ethanolamine-phosphate phospho-lyase	*AGXT2L1*	1.998	0.014	Fc receptor like 1	*FCRL1*	− 3.353	0.001
Cholesterol 25-hydroxylase	*CH25H*	1.858	0.001	Carbonic anhydrase 3	*CA3*	− 3.241	0.001
Interleukin 1 receptor antagonist	*IL1RN*	1.857	0.001	C-X-C motif chemokine ligand 13	*CXCL13*	− 3.136	0.030
Endothelial cell specific molecule 1	*ESM1*	1.850	0.009	CD79a molecule	*CD79A*	− 3.110	0.001
Phosphatase and actin regulator 3	*PHACTR3*	1.790	0.020	WNT inhibitory factor 1	*WIF1*	− 2.888	0.001
Lipase family member N	*LIPN*	1.783	0.022	Niban apoptosis regulator 3	*FAM129C*	− 2.867	0.001
TUB like protein 2	*TULP2*	1.703	0.031	Fc receptor like 3	*FCRL3*	− 2.776	0.001
Tudor domain containing 9	*TDRD9*	1.690	0.005	Cannabinoid receptor 2	*CNR2*	− 2.627	0.001
Activating transcription factor 3	*ATF3*	1.670	0.001	ADAM like decysin 1	*ADAMDEC1*	− 2.574	0.007
Chitinase 1	*CHIT1*	1.603	0.001	Fc receptor like 2	*FCRL2*	− 2.343	0.001
Solute carrier family 2 member 5	*SLC2A5*	1.580	0.001	Makorin ring finger protein 7, pseudogene	*MKRN7P*	− 2.318	0.037

We validated gene expression of select adipogenic, lipogenic, and inflammatory genes (*FASN,* peroxisome proliferator-activated receptor gamma (*PPARG),* CCAAT/enhancer-binding protein alpha *(CEBPA), CD34,* adiponectin *(ADIPOQ)*, leptin (*LEP),* glucose transporter type 4 (*GLUT4*), C–C motif chemokine ligand 18 (*CCL18*), cholesterol 25-hydroxylase (*CH25H*), C-X-C motif chemokine ligand 10 (*CXCL10*), endothelial protein C receptor (*EPCR*), lipopolysaccharide binding protein (*LBP*), matrix metalloproteases 2,7,8 (*MMP2*, *MMP7*, *MMP8*), osteopontin (*SPP1*), signal transducer and activator of transcription 5B (*STAT5B*), vitronectin (*VTN*)*)* identified as differentially regulated based on MS or RNASeq analysis in VAT samples from 8 NDM and 8 DM age- and BMI-matched female patients using quantitative real-time polymerase chain reaction (qRT-PCR) (Fig. 3b,c). We confirmed decreased expression of *FASN, PPARG, CEBPA, ADIPOQ*, and *GLUT4* in DM compared to NDM samples. No differences in inflammatory genes studied with qRTPCR reached significance.

### Transcriptional regulation of biological processes impacted by DM, similarities with proteomic analysis

RNA sequencing revealed 2373 GO terms significantly enriched before correction for multiple comparisons. The top significant biological processes identified by RNA sequencing analysis demonstrate a marked effect of DM on transcriptional regulation of innate and adaptive immune responses (Table 3). Immune response (GO: 0006955) and neutrophil degranulation (GO: 0043312) were the top two biological processes significantly regulated by DM, independent of pruning type. The top 20 DEG in each of the top biological processes are shown in Fig. 3d,e. Our data revealed 147 DEG out of all 1659 genes associated with immune response GO term, and 51 DEG out of all 448 associated with neutrophil degranulation GO term. Remarkably, 50 out of 51 DEG associated with neutrophil degranulation were upregulated in DM compared with NDM VAT samples (Fig. 3e); these genes play a key role in regulating exocytosis of neutrophil secretory granules. Additionally, proteomics and RNA sequencing analyses of human obese VAT revealed 13 shared biological processes significantly impacted by DM (Supplementary Table S6), primarily related to complement activation, cell proliferation and migration, ECM organization, lipid metabolism, and gluconeogenesis. In addition to FASN, other markers such as CFB (Fig. 3a), F13A1, thrombospondins (THBS4; Fig. 3a), and integrins were consistently regulated by DM at both mRNA and protein levels in human VAT.

**Table 3:** Top significant biological processes identified by RNA sequencing analysis of human VAT.

Pruning type^a^	None			Elim		Weight	
Biological process GO term	p-value	p-value (FDR)	p-value (Bonferroni)	Biological process GO term	p-value	Biological process GO term	p-value
Immune response	1.00E−24	1.00E−24	1.00E−24	Neutrophil degranulation	1.80E−19	Neutrophil degranulation	1.00E−20
Immune system process	1.00E−24	1.00E−24	1.00E−24	Inflammatory response	2.80E−12	Inflammatory response	3.40E−16
Defense response	1.00E−24	1.00E−24	1.00E−24	Regulation of complement activation	1.00E−10	Regulation of complement activation	2.10E−13
Positive regulation of immune system process	1.00E−24	1.00E−24	1.00E−24	Positive regulation of neutrophil chemotaxis	2.40E−09	Cytokine-mediated signaling pathway	1.90E−12
Inflammatory response	1.00E−24	1.00E−24	1.00E−24	Innate immune response	5.20E−09	B-cell receptor signaling pathway	7.40E−12

^a^*elim* and *weight* pruning methods were used to assess the enrichment of GO terms by considering the structure of the gene ontology. The *elim* pruning eliminated the genes mapped to a significant GO term from more general (higher level) GO terms, while the *weight* pruning assigned weight to each gene annotated to a GO term based on the scores of neighboring GO terms. The p-value GO terms was corrected for multiple comparisons using FDR and Bonferroni correction.

## Discussion

We demonstrate DM-specific VAT proteomic and transcriptomic profiles characterized by increased activity of pathways associated with inflammatory and humoral immune responses, complement activation, and cytoskeletal organization, and decreased mitochondrial function and lipid metabolic processes. Our findings have important commonalities with the few other published reports of the adipose tissue proteome in the context of obesity and DM (Supplementary Table S7) and provide insights into the role of VAT in DM pathogenesis.

Relative to fatty acid metabolism, we observed DM-specific decrease in VAT gene expression and protein abundance of FASN, a central lipogenic enzyme, accompanied by decreased gene expression of key regulators of adipogenesis and lipogenesis such as *PPARG, CEBPA, ADIPOQ,* and *GLUT4*. Multiple studies of transcriptomics and functional analyses of adipose tissue demonstrate defects in fatty acid synthesis in general and FASN specifically in obesity and DM^4,10–12^. Our proteome data revealed a decrease in enoyl-CoA hydratase and ethylmalonyl-CoA decarboxylase abundance in DM VAT, enzymes that catalyze early steps in fatty acid β-oxidation and lipid metabolite detoxification, levels of which have been shown to be decreased in subcutaneous adipose tissue (SAT) in obesity^13,14^, suggesting that defective metabolite detoxification capacity may exacerbate adipose tissue metabolic dysfunction in DM. Our transcriptome analysis also demonstrated downregulation of phospholipase A2 (*PLA2G4C*), which hydrolyzes glycerophospholipids to produce free fatty acids and lysophospholipids, and is involved in adipogenesis^15^. We also observed increased expression of *CH25H*, the 11th most upregulated gene in DM VAT compared to NDM. We recently reported that adipose tissue macrophages are the primary site of *CH25H* expression in humans and mice, and that obese *CH25H* knockout mice have improved insulin sensitivity and attenuated adipose tissue inflammation^16^. Together, these findings suggest that DM adipose tissue is characterized by global defects in lipid synthesis and fatty acid catabolism, a finding supported by prior functional studies demonstrating impaired adipocyte lipogenesis and fatty acid oxidation in DM^17–20^.

While we observed decreased levels of proteins involved in mitochondrial TCA cycle and oxidative phosphorylation/electron transport chain function in DM VAT, consistent with multiple prior data from proteomic^6–9,21,22^ and functional studies^23–27^, these findings were not evidenced by transcriptomics, suggesting that DM may regulate these processes at the post-transcriptional level. Specifically, proteomics revealed decreased abundance of the flavoprotein subunit of mitochondrial succinate dehydrogenase complex (*SDHA*) in DM, which links the TCA cycle, to mitochondrial electron transport. Others have implicated alterations in SDH in the adipocyte proteome: Benabdelkamel et al. found decreased levels of succinyl-CoA ligase in SAT from obese compared to lean subjects^14^, while Gomez-Serrano et al. revealed increased levels of succinate dehydrogenase cytochrome b560 in VAT adipocyte mitochondria from DM subjects, thought to be a compensatory response to down-regulation of other components of mitochondrial oxidative-phosphorylation complexes^21^. We also observed decreased protein abundance in DM VAT of dihydrolipoyllysine-residue acetyltransferase (DLAT) and DLD, the E2 and E3 components of the pyruvate dehydrogenase (PDH) complex. PDH catalyzes conversion of pyruvate to acetyl-CoA, linking glycolysis to the TCA cycle, and decreased PDH activity has been implicated in animal models of DM^28^. Other proteomics analyses demonstrate increased DLAT in murine brown compared to white adipocytes^29^, suggesting that defects at this point in the TCA cycle may be a commonality that distinguishes DM/NDM adipocytes and white/brown adipocytes. Together these data suggest that alterations in SDH and PDH function at the protein level may disproportionately contribute to disease-specific defects in adipocyte TCA and mitochondrial metabolism. Our proteomics data also revealed decreased levels of α and β subunits of the mitochondrial ATP synthase complex in DM VAT. Boden et al. demonstrated overexpression of mitochondrial ATP synthase in VAT from obese compared to lean subjects independent of DM, while Fang et al. observed decreased levels of mitochondrial ATP synthase coupling factor 6 in VAT from DM compared to NDM obese subjects^6,9^. Together these observations suggest the possibility that upregulation of ATP synthase may be a compensatory response in early obesity, while down-regulation may accompany progression to metabolic disease.

The top five biological processes regulated by DM in the VAT transcriptome in our data were associated with immune system and inflammatory response, processes previously implicated in metabolic syndrome^30,31^. Among the six proteins upregulated in DM VAT, vitronectin (VTN) and endothelial protein C receptor (EPCR) are directly associated with innate immunity. VTN is implicated in complement-based immune responses and inflammation and is elevated in serum of DM patients^32^, while EPCR is linked to DM nephropathy and vascular dysfunction^33,34^. We observed robust augmentation of complement components. VAT transcriptomics and proteomics shared 13 biological processes significantly affected by DM, four associated with complement activation and regulation. Previous studies suggest that activation of complement pathways plays a key role in inflammation in obesity^30,35^. In the present study, in addition to upregulation of *C2, C1q, C3, C4*, and complement factor B (*CFB*) genes, and increased protein abundance of C5, C6, C9 and CFB in DM VAT, our proteome data revealed expression of complement component C1q receptor in DM but not NDM VAT. Increased expression of C1 complement components by adipocytes is associated with induction of insulin resistance in vitro, in animal models of obesity, and in insulin-resistant subjects^30^, and activation of classical C1q and components of alternative complement system has been shown to induce adipose tissue inflammation in obese, insulin resistant subjects^31^. Another study demonstrated that complement components C2, C3, C4, C7, and CFB were expressed at higher levels in VAT compared with SAT^36^, suggesting unique dysfunction of the complement system in visceral adiposity. Our data demonstrate increased CFB gene expression and protein abundance in DM VAT. CFB is elevated in adipose tissue and serum of patients with DM and cardiovascular disease, and abundantly expressed in inflamed adipose tissue^37,38^. *Cfb*^*−/−*^ rats showed improved glucose tolerance and insulin sensitivity, redistribution of visceral to subcutaneous fat, and increased adipocyte mitochondrial respiration^37^. Additionally, CFB secretion by macrophages is increased in a murine model of severe inflammation^39^ and linked to proliferation and differentiation of preactivated B-lymphocytes and rapid spreading of peripheral blood monocytes, mechanisms implicated in adipose tissue inflammation in humans^40^. Together, these data suggest that elevated expression of complement system components plays an important role in VAT dysfunction in human DM.

Our transcriptomic and proteomic data demonstrate increased activity of pathways involved in cytoskeletal function, and cell proliferation and migration, mainly related to leukocyte function. There was a consistent impact of DM on VAT gene expression and protein abundance of integrins (ITGB1, ITGAM, ITGB2), thrombospondins (THBS1, THBS4), and F13A2, proteins involved in cell migration and proliferation, ECM organization, and platelet degranulation, findings consistent with previous studies^14,22^. A hallmark of adipose tissue remodeling in obesity is induction of matricellular proteins, such as thrombospondins and osteopontin (SPP1), which regulate inflammatory, reparative, fibrogenic, and angiogenic responses^41^. Consistent with our findings, THBS1 is overexpressed at protein and mRNA levels in adipose tissue from obese humans^42,43^, with expression levels associated with inflammation and metabolic dysfunction^44^. Notably, our transcriptomics data revealed downregulation of *THBS4* in DM, a protein that promotes angiogenesis and reduces collagen production^45^, features related to adipose tissue remodeling in the context of obesity. Finally, we observed marked upregulation of genes associated with immune cell migration and proliferation (C-X-C motif chemokine ligand 10, *CXCL10; Chemokine ligand 13, CCL13*), specially related with T-cell function (e.g. *CD28, CD4, CD86, CCL24*), which is demonstrated by us and others to play an essential role in modulating adipose tissue inflammation in obesity^46,47^.

We studied VAT because visceral adiposity is closely associated with metabolic disease, and we restricted analysis to females to eliminate confounding effects of sex on results; future analyses will be necessary to identify depot-specific targets and sex differences in the adipose tissue proteome. We matched DM and NDM groups for age and BMI, but this study is underpowered to control for confounding effects of medications and other metabolic diseases, which cannot be ruled out as contributing to observed differences, an inherent weakness of all human studies that will require larger cohorts to address. Nonetheless, our criteria for defining DM and NDM subjects were stringent, supporting DM as the most likely clinical correlate of observed differences. We studied whole adipose tissue, in contrast to other reports, some of which study isolated adipocytes, stromovascular cells, or mitochondria, in order to identify targets in all adipose tissue cell types. Nonetheless, commonalities of our results with prior reports studying other cell fractions suggest robust disease associations. Only a few specific proteins identified by our study were identified by similar studies; lack of overlap of identified targets among proteomics studies has been demonstrated before^9^, likely due to differences in human subjects, tissues and cells studied, protein preparation, and MS methodology. Nonetheless, strong concordance between our study and others with respect to implication of mitochondrial, TCA, inflammatory, and cytoskeletal pathways supports clinically relevant findings. Furthermore, the few specific targets we identified that overlap with other studies (e.g. FASN, CFB, C1qR, THBS-1 and 4, SDH, DLAT, ATP synthase) suggest high yield targets. Of three proteins tested with Western blot, we observed statistically significant validation of differential expression for FASN and SDH-α, but not DLD. Fold differences between DM and NDM groups for most targets based on proteomics and were modest, ~ twofold, differences that may not be detectable with Western blotting. Future study of isolated subcellular fractions (e.g. mitochondrial isolates) may provide greater sensitivity. Similarly, qRTPCR did not confirm DEG of inflammatory targets, suggesting that suboptimal sensitivity of PCR for modest differences detected by RNASeq, but pathway analysis nonetheless confirmed disruption of inflammatory signaling, consistent with multiple prior reports. Higher levels of stringency in identifying specific DEG will be required in future research focusing on specific gene targets that stem from this discovery analysis. This study did not have sufficient depth of coverage or include an enrichment procedure that would have made analysis of post-translational modifications of proteins possible. Such analysis is beyond the scope of this discovery analysis, but will be an important component of future research. The human diabetes-specific visceral adipose tissue proteome and transcriptome are characterized by dysregulation of pathways involving lipid metabolism, inflammation and innate immunity, ECM/cytoskeleton function, cell migration, TCA cycle, and mitochondrial function. Both proteomic and RNA sequencing analyses of human obese VAT identify decreased fatty acid synthase expression as strongly associated with DM. This is one of only a few studies that reports combined transcriptomic and proteomic profiles of adipose tissue in obesity, and the only study of which we are aware that links these findings to DM. These data provide insight into the mechanisms of adipose tissue dysfunction in DM and suggest targets for further study.

## Methods

### Ethics statement

Human subjects research was performed with approval from Institutional Review Boards at the University of Michigan and Veterans Affairs Ann Arbor Healthcare System under ethics guidelines consistent with in accordance with ethical standards laid down in the 1964 Declaration of Helsinki and the 1974 Belmont Report.

### Human subjects

Human subjects provided voluntary informed consent for study participation with approval from Institutional Review Boards at the University of Michigan and Veterans Affairs Ann Arbor Healthcare System in accordance with ethical standards for informed consent outlined in the 1974 Belmont Report. Visceral adipose tissue (VAT) from the greater omentum was collected from obese diabetic (DM) and non-diabetic (NDM) women during bariatric surgery, and snap-frozen and stored at − 80 °C until further processing as outlined below. DM was defined by clinical diagnosis requiring medication and hemoglobinA1c (HbA1c) ≥ 6.5%. Non-diabetic (NDM) subjects were defined by no clinical history of diabetes and HbA1c < 5.7% per American Diabetes Association criteria^48^ (Supplementary Table S8).

### Human VAT preparation for mass spectrometry

VAT (200 mg) samples were homogenized at 4 °C in 200 µl 40 mM Tris pH 8, 5 mM dithiothreitol using a bullet blender. Protein and lipids were separated by adding 800 µl methanol/chloroform (2:1 v:v) and centrifuging. Protein was extracted and washed with 750 µl of methanol/chloroform (2:1 v:v), dried with a speed-vac, solubilized in 40 mM Tris pH 8, 2% sodium deoxycholate (DOC), and total protein concentration measured using BCA assay (Thermo Fisher Scientific Inc., Waltham, MA, USA). Proteins were heat denatured 95 °C, 5 min, reduced with DTT, alkylated with iodoacetamide, diluted to 1% DOC and trypsinized. DOC was precipitated with formic acid prior to MS analysis. Protein abundance was measured using label-free proteomic analysis with a ThermoFusion tribrid mass spectrometer (Thermo Fisher Scientific Inc., Waltham, MA, USA) equipped with an easy nLC and 50 cm easy-spray reversed phase column using 140 min LC gradients, set to collect MS1 scans at 120 K resolution. Concurrent data-dependent MS2 scans were collected in an ion trap analyzer at 1 s/cycle.

### Proteomic data processing and statistical analysis

Data were searched using Maxquant v1.6.1.0 against a human complete database downloaded from Uniprot on 2017.07.14 containing 20,145 entries. Match between runs was enabled and proteins required only 1 peptide for quantification and both modified and unmodified peptides were used for quantification. All other settings were default values. Data analysis was carried out in R v3.1.3. Protein abundances were normalized such that each sample had the same median. Proteins quantified in fewer than 4 samples in either group were analyzed using a heuristic approach to identify those below limit of detection in one group but not the other. Proteins with 4 or more quantitative values for both DM and NDM samples were analyzed for differences between groups using a moderated t-test with missing value imputation. Imputed values were chosen for DM and NDM samples separately from a normal distribution with the same mean and standard deviation as the present data for each protein. Between group differences were identified using moderated linear regression^49^ with disease (DM/NDM) and sample preparation batch as predictors. A q-value was calculated for each protein to account for multiple testing^50^. Imputations were repeated 50 times. Proteins that were significant at a 10% false discovery rate (q < 0.1) in at least 30 imputations were considered to be different between groups. Biological processes and cellular components (GO categories^51^) differentially regulated between DM and NDM tissues were identified by PIANO software^52^, using a set-based approach that considers change in abundance of all proteins in a given category collectively without regard to which proteins pass a statistical threshold. This analysis uses t-statistics from moderated t-test and category enrichment determined through mean t-statistic. To reduce redundancy, biological processes were clustered, with the lowest p-value in each cluster reported. Clustering was performed to group redundant GO categories and just the most strongly affected category from each cluster was reported. Clusters with a minimum of 3 nodes were included. Protein membership overlap between categories and dynamic tree cut algorithm^53^ was used for clustering.

### Western blotting

50 μg of VAT protein lysates were run on a 4–20% gradient gel then transferred to PVDF membrane (Bio-Rad Laboratories Inc., Hercules, CA, USA). Blots were blocked for 1 h, 25 °C in blocking buffer (PBS, 5% BSA, 0.5% Tween-20), stained with primary antibodies in blocking buffer overnight, 4 °C, washed in wash buffer (PBS 0.1% Tween-20), then actin-specific antibody applied for 2 h, 25 °C in blocking buffer. Next, secondary HRP-conjugated antibodies were applied for 1 h, 25 °C in blocking buffer. Blots were imaged on an Azure 600 Western Blot Imaging System using HRP substrate Super Signal West Pico Plus (Thermo Fisher Scientific Inc., Waltham, MA, USA), and densitometry performed with ImageJ software gel analysis tools (http://lukemiller.org/index.php/2010/11/analyzing-gels-and-western-blots-with-image-j, San Jose State University, San Jose, CA, USA). Band density values between separate blots were normalized to a 50 μg referent protein sample comprised of a 50:50 mixture of one DM sample and one NDM sample, then normalized by densitometry values between samples to matched β-actin loading controls, using the following antibodies: DLD (Novus Biologicals Inc., Littleton, CO, USA, Cat#NBP2-19361, 1:3000); SDH-α (Thermo Fisher Scientific Inc., Waltham, MA, USA, Cat#PA5-79964, 1:3000); FASN (Cell Signaling Inc., Danvers, MA, USA, Cat#3189S, 1:1000); β-Actin (Invitrogen Inc., Waltham, MA, USA, Cat#MA5-15739, 1:10,000), anti-rabbit HRP (Sigma-Aldrich, St. Louis, MO, USA Cat#AP132P, 1:10,000) and anti-mouse HRP (Thermo Fisher Scientific Inc., Waltham, MA, USA, Cat#31430, 1:10,000). Statistical comparison of protein expression between DM and NDM groups was performed using unpaired two-sided t-tests using GraphPad Prism 8 software, with p < 0.05 considered significant.

### RNA sequencing

Total RNA was extracted (Qiagen Inc., Hilden, Germany), polyA enriched, and subjected to RNA sequencing using Illumina Hi-Seq platform (> 80 reads/sample). Data analysis used FastQC (v0.10.0) and Tuxedo Suite package for alignment, differential expression analysis, and post-analysis diagnostics aligned to the UCSC hg19 reference genome using TopHat, Bowtie, and Cufflinks/CuffDiff. Data visualization and pathway analysis was performed with iPathwayGuide (Advaita Corp., Ann Arbor, MI, USA). Differentially expressed genes (DEG) were identified using thresholds of 0.05 for statistical significance (p-value) and log-fold change of expression of at least 0.5. DEG were analyzed in the context of pathways from the Kyoto Encyclopedia of Genes and Genomes (KEGG) database (Release 90.0 + /05–29, May 19)^54^ and gene ontologies (GO) from the Gene Ontology Consortium database (2019-Apr26)^55^. For each GO term, the number of DEG annotated to the term was compared with numbers of DEG expected by chance. iPathwayGuide uses an over-representation approach to compute statistical significance of observing at least the given number of genes, considering all GO terms to be independent. Enrichment of GO terms were assessed by *elim* and *weight* pruning methods^56^. The *elim* pruning method iteratively eliminates the genes mapped to a significant GO term from more general (higher level) GO terms, while the *weight* pruning method assigns weight to each gene annotated to a GO term based on scores of neighboring GO terms. p-value GO terms were corrected for multiple comparisons using FDR and Bonferroni.

### qRT-PCR

RNA extraction and qRT-PCR were performed as described^3^. Human VAT was lysed in Trizol and RNA extracted with RNAEasy Fibrous Tissue MiniKit (Qiagen Inc., Hilden, Germany). Equal amounts of input RNA were reverse transcribed using Applied Biosystems High Capacity cDNA Archive Kit (Applied Biosystems Inc., Foster City, CA, USA). qRT-PCR was conducted with TaqMan primers and reagents (Life Technologies Inc., Carlsbad, CA, USA). Data are presented as fold change of DM relative to NDM samples calculated from least squares mean differences according to 2^−ΔΔCt^ method^57^, normalized to mean *B2M* expression.

### Methods statement

All methods were carried out in accordance with relevant guidelines and regulations as outlined in the Springer Nature website. Human subjects provided informed consent and were enrolled with approval from Institutional Review Boards at the University of Michigan and Veterans Affairs Ann Arbor Healthcare System. Enrollment, consent, and all aspects of human subjects research were carried out in accordance with the Belmont Report from the National Research Act of 1974, and the Declaration of Helsinki set forth by the World Medical Association. This manuscript contains no human participants' names or other HIPAA identifiers.

## Supplementary Information

Supplementary Table S1.

Supplementary Table S2.

Supplementary Table S3.

Supplementary Table S4.

Supplementary Table S5.

Supplementary Table S6.

Supplementary Table S7.

Supplementary Table S8.

## Data Availability

Proteomics data are available in the ProteomeXchange (http://www.proteomexchange.org/), dataset PXD021147. All data generated and analyzed during the current study are included in the published article and its supplementary information files. All reagents will be freely provided upon reasonable request, except for human tissue and cell samples, and human subject clinical information or identifying information, which are not permitted to be shared due to IRB, HIPAA, and confidentiality constraints.

## Abbreviations

BMI: Body mass index
DEG: Differentially expressed genes
DM: Type 2 diabetes/diabetic
ECM: Extracellular matrix
HbA1c: Hemoglobin A1c
MS: Mass spectrometry
LC–MS: Nano-liquid chromatography-mass spectrometry
NDM: Non-diabetic
qRT-PCR: Quantitative real-time polymerase chain reaction
SAT: Subcutaneous adipose tissue
TCA: Tricarboxylic acid cycle
VAT: Visceral adipose tissue
