# Supplementary material for: The human type 2 diabetes-specific visceral adipose tissue proteome and transcriptome in obesity
Source: Sci Rep. 2021 Aug 30;11:17394. doi: 10.1038/s41598-021-96995-0 (PMC8405693; doi:10.1038/s41598-021-96995-0)
Supplement: Supplementary file 6 — Supplementary Table S6. [file 41598_2021_96995_MOESM6_ESM.docx]

**Supplementary Table S6.** Common differentially regulated biological processes identified by both proteomic analysis and RNA sequencing of VAT. Table shows differentially abundant proteins and DEG in DM relative to NDM patients. Differentially regulated genes (DEG) for specific biological processes are shown as genes with enriched or downregulated expression in DM relative to NDM samples.

| **Biological process category** | **Identified by proteomics** | | **Identified by RNA sequencing** | | | | |
| --- | --- | --- | --- | --- | --- | --- | --- |
|  | **p-value** | **Affected Proteins** | **# DEG/all*** | **p-value** | **Genes enriched in DM/NDM** | **Genes downregulated in DM/NDM** |  |
| Regulation of complement activation | 2.12E-11 | VTN, C5, C6, C9, CFB | 14 / 36 | 4.10E-14 | *C2, C1QC, C1QA, CFB, CD55, CR1, C5AR1, C4BPB, C3AR1, VSIG4, CFP, C1QB* | *CR2, CD19* |  |
| Complement activation, alternative pathway | 6.24E-07 | C5, C9, CFB | 4 / 9 | 3.80E-05 | *CFB, CR1, CFP, VSIG4* | *-* |  |
| Complement activation | 1.09E-04 | C5, C6, CFB | 15 / 45 | 7.20E-14 | *C2, C1QC, C1QA, CFB, CD55, CR1, C5AR1, C4BPB, C3AR1, FCN1, VSIG4, CFP, C1QB* | *CR2, CD19* |  |
| Complement activation, classical pathway | 1.09E-04 | C5, C6, C9 | 8 / 23 | 3.80E-08 | *C2, C1QC, C1QA, C4BPB, CD55, CR1, C1QB* | *CR2, CD19* |  |
| Negative regulation of endopeptidase activity | 4.31E-03 | VTN, C5 | 9 / 151 | 0.011 | *CR1, MMP9, PTGS2, SLPI* | *COL4A3, CPAMD8, IFI6, LEF1, SPOCK1* |  |
| Positive regulation of angiogenesis | 4.31E-03 | C5, C6 | 12 / 171 | 9.10E-04 | *C3AR1, C5AR1, CCL24, CHI3L1, CXCL8, CYBB, HMOX1, ITGB2, PTGS2* | *APLNR, GREM1, HSPB6* |  |
| Platelet degranulation | 2.01E-04 | F13A1, PLG, THBS1 | 8 / 114 | 0.006 | *F13A1, FCER1G, FN1, PLEK, SERPINA1, SRGN, SYK* | *TF* |  |
| Cell migration | 7.04E-03 | ITGB1, THBS1 | 63 / 1192 | 1.90E-09 | *AIF1, APOE, APOE, C3AR, C4BPB, C5AR1, CCL13, CCL24, CCL3, CCL4, CCR1, CH25H, CMKLR1, CSF1R, CSF3R, CXCL10, CXCL8, DOK2, F13A1, FAT2, FCER1G, FGR, FN1, GNG2, HCK, HMOX1, ITGAM, ITGB2, JUN, LBP, LCP1, LEP, LGMN, MERTK, MMP9, MYOC, NCKAP1L, NR4A1, NR4A2, NR4A3, PIK3R5, PLEK, PTGS2, PTPRO, S100A8, S100A9, SELPLG, SERPINA1, SIX1, SLC7A7, SYK, TREM1, TREM2* | *ADGRL3, AKAP1, CCL19, CCL21, CCR7, CEMIP, CNR2, COMP, CXCL13, EPPK1, GPM6A, GREM1, HBG2, LEF1, MIR34A, MYOCD, NTRK3, RELN, SPOCK1, THBS4* |  |
| Blood coagulation | 7.04E-03 | F13A1, PLG | 13 / 278 | 0.018 | *APOE, C4BPB, F13A1, FCER1G, GNG2, MERTK, PIK3R5, PLEK, SERPINA1, SYK* | *AKAP1, COMP, HBG2* |  |
| Extracellular matrix organization | 7.04E-03 | ITGB1, THBS1 | 19 / 290 | 8.10E-05 | *CTSL, CTSS, FBN2, FN1, ITGAM, ITGB2, LCP1, MFAP5, MMP7, MMP8, MMP9, SPP1* | *AEBP1, COL4A3, COMP, GREM1, ITGA8, MYH11, NPNT* |  |
| Positive regulation of cell proliferation | 7.04E-03 | ITGB1, THBS1 | 44 / 749 | 3.80E-08 | *AIF1, ATF3, C5AR1, CCL24, CCND1, CD209, CD28, CD4, CD55, CD86, CDKN1A, CSF1R, CTSZ, CXCL10, EGR1, ESM1, FN1, FOLR2, HAVCR2, HCK, HLA-DMB, HMOX1, IL2RA, JUN, LEP, LGMN, LRP2, MMP9, NCKAP1L, NR4A1, NR4A3, PTGS2, SIX1* | *APLNR, CCL19, CNTFR, FCRL3, GREM1, IL7R, LEF1, MYOCD, NTRK3, THBS4, TNFRSF13C* |  |
| Lipid metabolic process | 1.06E-03 | ATP5B, ATP5A1 | 43 / 1149 | 0.003 | *ALOX5AP, APOE, B4GALT6, CES1, CH25H, CSF1R, DGAT2, DHRS9, EGR1, ETNPPL, FGR, FUCA1, GPAT2, HPGDS, IL1RN, LEP, LGMN, LIPA, LIPN, LRP2, NCEH1, NR4A3, PIK3R5, PLA2G15, PLA2G2A, PLBD1, PLEK, PTGS2, SCD, SIK1, SOCS3, SPP1, TBXAS1, TIPARP* | *CACNA1H, CCL19, CCL21, CCR7, CD19, CIDEA, FASN, PDE3A, PLA2G4C* |  |
| Gluconeogenesis | 6.07E-04 | MDH2, GPD1 | 6 / 78 | 0.011 | *ATF3, DGAT2, FBP1, LEP, MDH1, SIK1* | *NA* |  |

* #DEG/all refers to the number of genes differentially regulated out the total number of genes in that specific biological process.
