# Supplementary material for: The human type 2 diabetes-specific visceral adipose tissue proteome and transcriptome in obesity
Source: Sci Rep. 2021 Aug 30;11:17394. doi: 10.1038/s41598-021-96995-0 (PMC8405693; doi:10.1038/s41598-021-96995-0)
Supplement: Supplementary file 8 — Supplementary Table S8. [file 41598_2021_96995_MOESM8_ESM.docx]

**Supplementary Table S8.** Demographic and clinical information of female obese patients with (DM) and without (NDM) type 2 diabetes included in this study

|  | | **Proteomics** | | | **Western blotting/**  **qRT-PCR** | | | **RNA sequencing** | | |
| --- | --- | --- | --- | --- | --- | --- | --- | --- | --- | --- |
|  |  | **NDM (n=10)** | **DM (n=10)** | **p-value** | **NDM (n=8)** | **DM (n=8)** | **p-value** | **NDM (n=5)** | **DM (n=5)** | **p-value** |
| Subjects demographics | Age (mean ± SD, years) | 39 ± 11 | 42 ± 10 | 0.693 | 49 ± 6 | 52 ± 10 | 0.627 | 45 ± 5 | 44 ± 8 | 0.788 |
|  | BMI (mean± SD, kg/m^2^) | 45 ± 7 | 44 ± 6 | 0.690 | 42 ± 6 | 46 ± 7 | 0.324 | 42 ± 7 | 45 ± 5 | 0.498 |
|  | HbA1c (mean) | 5.40% | 6.90% | <0.001 | 5.40% | 6.70% | 0.013 | 5.04% | 7.32% | 0.001 |
| Comorbid diseases (% of patients) | Sleep apnea | 70% | 60% | 0.660 | 50% | 71% | 0.608 | 80% | 80% | >0.999 |
|  | Hypertension | 20% | 50% | 0.121 | 0% | 43% | 0.077 | 60% | 80% | 0.523 |
|  | Dyslipidemia | 10% | 30% | 0.288 | 13% | 57% | 0.119 | 60% | 60% | >0.999 |
| Medications (% of patients) | ACE inhibitor | 0% | 44% | 0.022 | 0% | 0% | >0.999 | 0% | 60% | >0.999 |
|  | Beta-blocker | 0% | 22% | 0.110 | 13% | 17% | >0.999 | 0% | 0% | >0.999 |
|  | Insulin | 0% | 56% | 0.006 | 0% | 0% | >0.999 | 0% | 0% | >0.999 |
|  | Metformin | 0% | 78% | <0.001 | 0% | 100% | 0.0003 | 0% | 100% | 0.007 |
|  | Statin | 0% | 44% | 0.022 | 0% | 33% | 0.165 | 0% | 20% | >0.999 |
|  | Sulfonylurea | 0% | 22% | 0.110 | 0% | 33% | 0.165 | 0% | 0% | >0.999 |
|  | Thiazolidinedione | 0% | 0% | >0.999 | 0% | 0% | >0.999 | 0% | 0% | >0.999 |
|  | GLP-1 agonist | 0% | 22% | 0.110 | 0% | 0% | >0.999 | 0% | 0% | >0.999 |
|  | DPP-4 Inhibitor | 0% | 22% | 0.110 | 0% | 0% | >0.999 | 0% | 0% | >0.999 |

Independent t-test was used to compare continuous variables between DM and NDM groups; Fisher’s exact test used to compare dichotomous variables between DM and NDM groups; SD: standard deviation
